# Diamonds in the rough - reconsidering the scientific and heritage value of heat-altered stones in prehistoric archaeology through a systematic literature review

**DOI:** 10.12688/openreseurope.18837.1

**Published:** 2024-11-27

**Authors:** Margherita Cantelli, Xavier Terradas, Didier Binder, Martine Regert, André Carlo Colonese

**Affiliations:** 1 Institute of Environmental Science and Technology (ICTA-UAB) and Department of Prehistory, Universitat Autonoma de Barcelona, Barcelona, 08193, Spain; 2 CNRS, CEMPAM, Universite Cote d'Azur, Nice, 06300, France; 3 Spanish National Research Council (IMF-CSIC), Barcelona, 08001, Spain

**Keywords:** Heat altered stones (HAS); prehistoric archaeology; culinary practices; systematic literature review; archaeological research methods

## Abstract

**Background:**

Heat-altered stones (HAS) are commonly reported in prehistoric sites across several continents, yet they continue to be generally overlooked and systematic studies on them are scarce.

**Methods:**

We performed a systematic literature review which consisted of searching journal articles, book chapters and books published in English, in Scopus and Web of Science. We collected information on their geographic distribution, chronology, composition, technological aspects and subsistence contexts from 73 records. Our aims were to assess the challenges and opportunities of analysing HAS, while highlighting that this class of artefacts is still largely an untapped source of information on prehistoric human activities.

**Results:**

HAS have been documented since the Pleistocene, attesting that culinary and non-culinary activities using heating stones emerged among foraging groups subsisting on hunting, fishing and gathering. The high frequency of HAS during the middle and late Holocene testifies to the continuation of some practices over long time periods, amid the emergence of new food systems, and the introduction of new resources and technologies, such as domesticated plants and animals, and ceramic containers. A considerable lack of studies on HAS from Africa, Oceania, Asia, and South America was noted, all of which are key geographic areas for assessing the role of heating stones in human evolution, geographic dispersal, early cuisine and diet, and cultural transmission across the globe.

**Conclusions:**

Our results highlight the persistent challenges archaeologists face in establishing fundamental definitions and diagnostic criteria for identifying HAS, while emphasizing the importance of HAS as essential elements for studying ancient foodways and cultural heritage. We call on archaeologists and cultural heritage managers to reconsider the heritage value of HAS and include them in specialised research agendas before significant knowledge of our past is lost.

## Plain Language Summary

This study examines heat-altered stones (HAS), namely rocks that were heated and used by past people and found abundantly at archaeological sites. These stones offer valuable insights into the lifeways of past societies, including their cooking practices, diet, and use of fireplaces. HAS have been recovered in sites dating back to the Pleistocene, suggesting that even before the advent of farming and herding, people were heating stones for cooking and other activities. HAS continued to be used for thousands of years, evolving alongside the adoption of new tools and food systems. Although archaeologists have often found, recorded, and sometimes collected these stones, in-depth studies of HAS remain rare. Here we compiled existing knowledge on HAS, exploring their geographic and chronological origins, their uses, and how scholars have analysed them. Much remains unknown, especially concerning HAS from Africa, Oceania, Asia, and South America. Expanding research to include these regions can deepen our understanding of how ancient cultures used fire, prepared food, and shared these practices globally.

## Introduction

Food, as a social phenomenon and as subsistence, is characterised by intricate operational chains encompassing the acquisition, distribution, transformation, storage and consumption of natural resources, including medicinal and symbolic considerations. The ways in which food is prepared and consumed provide profound insights into the collective identity of communities, serving as a vehicle of power, rules, communication, traditions and social dynamics ( Graff, 2020; Hastorf, 2016; Morrison, 2012). Culinary practices are closely connected to the labour and life of the individuals involved in the gathering, handling, storage, processing, cooking, and consumption of the food ( Graff, 2018; Morrison, 2012; Twiss, 2012). Such complexity between food and culture has roots that reach back to prehistoric times, where the role of food was pivotal in human evolution and social organisation ( Graff, 2020; Twiss, 2012).

Foodways have been a focal point in archaeological research for several decades, as evidence of cooking and food preparation can unveil crucial insights into the life of past human groups ( Graff, 2020). Profound changes in food production and preparation techniques have been documented in prehistoric times and in some cases they have helped to define key cultural changes, such as the adoption of agriculture and storage technology ( Fuller & Carretero, 2018; Griffin, 1967). Along with the control of fire, one of the most significant evolutionary changes in prehistoric food preparation techniques was the adoption of ceramic artefacts for manipulating, boiling, storing, and fermenting a variety of foodstuffs and other substances ( Craig, 2021). Pottery use in prehistoric households is thought to have improved palatability and digestibility of food items, thus expanding diet and creating more complex and varied meals that laid the foundation of culinary traditions in the past ( Fuller & Carretero, 2018). In fact, the consumption of food was not limited to raw ingredients found in nature but involved the transformation of natural resources into cultural products through alternative techniques of food processing and cooking, such as grinding, roasting, baking, boiling, fermenting, and mixing. These techniques enhanced the edibility, nutritional value, and storability of food, as well as influenced their sensory qualities and taste ( Carmody & Wrangham, 2009; Kubiak-Martens, 2002; Morrison, 2012). Functional properties of edible foods are crucial to comprehend the connections between dietary composition and the technological decisions made by past human groups ( Morrison, 2012; Wollstonecroft, 2011).

Prehistoric cooking technology evolved through time and space in different ways, adopting a certain diversity according to the environment and the availability of resources. However, our understanding of this process is hampered by the poor preservation of organic materials and often biased towards a handful of archaeological artefacts including primarily ceramics and grinding stones. Other long-lasting cooking solutions have only received cursory attention, as is the case of boiling and roasting food with the aid of stones (exposed to high temperatures ( Kipfer, 2023)), of which evidence is alluded to by the presence of Heat Altered Stone (HAS) in prehistoric sites. HAS are generally associated with household contexts, combustion structures and cooking activities ( Custer, 2017; Frère-Sautot, 2003; Gaugler, 2019; Gerritsen *et al.*, 2013; Graesch *et al.*, 2014; Hart, 2023; Kelley & Campbell, 1942; Neubauer, 2018; Petersson, 2013; Petraglia, 2002; Stuart & Walker, 2018), for which they are repeatedly heated, reused, and eventually discarded somewhere nearby, in a sort of toss zone ( Black & Thoms, 2015; Fishel *et al.*, 2003; Leesch *et al.*, 2010; Nakazawa *et al.*, 2009; Neubauer, 2018; Sullivan *et al.*, 2001). These stones are believed to be used as heating elements in a variety of cooking methods, and possess the physical properties to capture, retain and dissipate heat. Nevertheless, a range of non-culinary activities such as sweat lodge rituals ( Barfield & Hodder, 1987; Custer, 2017) and the heating of domestic and public spaces ( Hawkes, 2018; Svoboda, 2008), may similarly expose stones to high temperatures, complicating the identification and systematic study of HAS in the archaeological record. In addition, considerable uncertainty still remains about their geographic, chronological and cultural distribution, as well as their relation to particular resources and other food processing techniques ( Graesch *et al.*, 2014; Petraglia, 2002).

Over the years, HAS have been documented by archaeologists without initially being a central research focus. This trend started to shift during the 1970s and 1980s, with growing attention on understanding fire-related activities. In Europe, for instance, modern research on how prehistoric spaces were structured is heavily influenced by the school of A. Leroi-Gourhan ( Leroi-Gourhan & Brézillon, 1966; Leroi-Gourhan & Brézillon, 1972), which gave birth to the new field of comparative technology using analytical methods for comparing technical aspects. Consequently, the research started to move beyond simply describing hearths to investigating their role and use in prehistoric life. Similarly, Binford’s research ( Binford, 1978) provided a framework for understanding the various ways fire can be used, offering new insights into fire-related archaeological evidence. Within this context HAS were not studied alone, but as part of the bigger picture of fire structures use and the organisation of space around them. They have typically been presented in monographs, conference proceedings and excavation reports as components of various structures ( Binder *et al.*, 1991; Frère-Sautot, 2003; Olive & Taborin, 1989), indicating they were not given comprehensive consideration. As a result, finding detailed studies on HAS in published records is challenging.

To address some of these gaps we conducted a review of the global literature reporting on archaeological HAS. Systematic literature reviews are essential parts of academic research ( Linnenluecke *et al.*, 2020; Nightingale, 2009; Okoli, 2015; Xiao & Watson, 2019), through which a starting point for larger research can be provided. We examined 71 scientific articles, book chapters and books from 1942 to 2023, and compiled information to derive a comprehensive perspective on the geographic distribution, chronology, technological, subsistence and culinary contexts of HAS across different regions and time. We assessed some of the analytical advances and main challenges of studying HAS over the last decades.

## Bibliographic survey and database construction

We performed a systematic literature review in April 2024, which consisted of searching journal articles, book chapters and books published in English, in Scopus and Web of Science. In both databases ALL FIELDS were searched using specific keywords relevant to the scope of this work: “Stone cooking” AND “Archaeology”, OR “Fire-cracked stones” AND “Archaeology”, OR “Heat altered stones” AND “Archaeology”, OR “Boiling stones” AND “Archaeology”. A total of 46 publications (items) were obtained from Scopus and 82 from the Web of Science. Duplicates were then removed, resulting in 123 items. These items were then analysed as follows, to take into account possible subject bias. Firstly, the abstract of each item was read independently by the first and the last authors to evaluate the relevance based on the inclusion criteria “stone/rock(s)” related to cooking/culinary activities. The first and last authors then discussed their independent assessments, which resulted in the exclusion of 84 items, reducing the dataset to 39 items. Secondly, the items were fully read by the two authors to ascertain the relevance of the entire research and a total of 5 items were excluded due to their focus on groundstones, ceramics and flake production, which were deemed off-topic. The final number of eligible items was 34. The systematic literature review, however, failed to capture several research outputs that were cited in the selected items. This prompted us to consider a snowball sampling method ( Wohlin *et al.*, 2022) to increase the overall record, which increased the number of items to 73. Grey literature (such as master's dissertations, PhD theses, and local excavation reports) was excluded from our search due to the unpredictability of mapping these resources on a global scale. Although this type of literature can provide valuable information, conducting research on global grey literature would be infeasible.

The 73 items were distinguished between experimental study (including modelling) and/or archaeological reports, and catalogued in an Excel file, with columns containing information about a set of systematically collected variables (Extended data). Descriptive and binary variables related to contextual information included region, site, cultural attribution (e.g. Neolithic, Middle Woodland, etc.), chronology (as reported in the original publications), subsistence system (foraging, farming, foraging/farming), presence of ceramics (Y/N), if stones were found as part of a structure(s) (hearth, stone piles, stone pits), etc. These were followed by the terminology employed to describe the stones, mineral composition and type of analysis. In addition, we classified the items according to the main content of the study: 1) identification and characterization of heat altered stones, 2) interpretation of stone use (cooking, heating facilities), 3) theoretical propositions on the evolving nature of these stones and relative social implications. 

## Literature results

**Figure 1.**
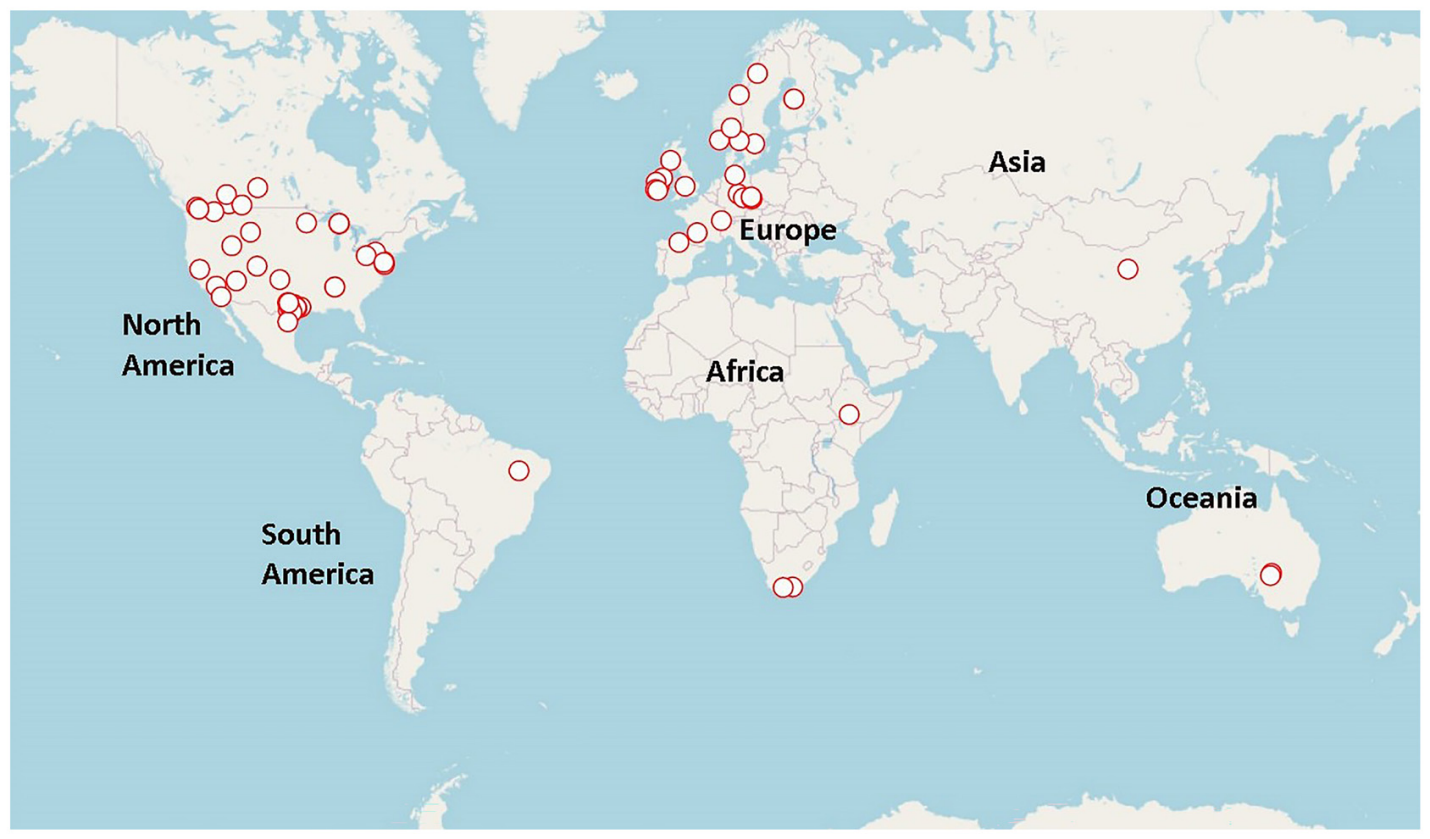
Geographical location of the case studies (the world map was created using the OpenStreetMap package: Fellows & Stotz, 2023).

**Figure 2.**
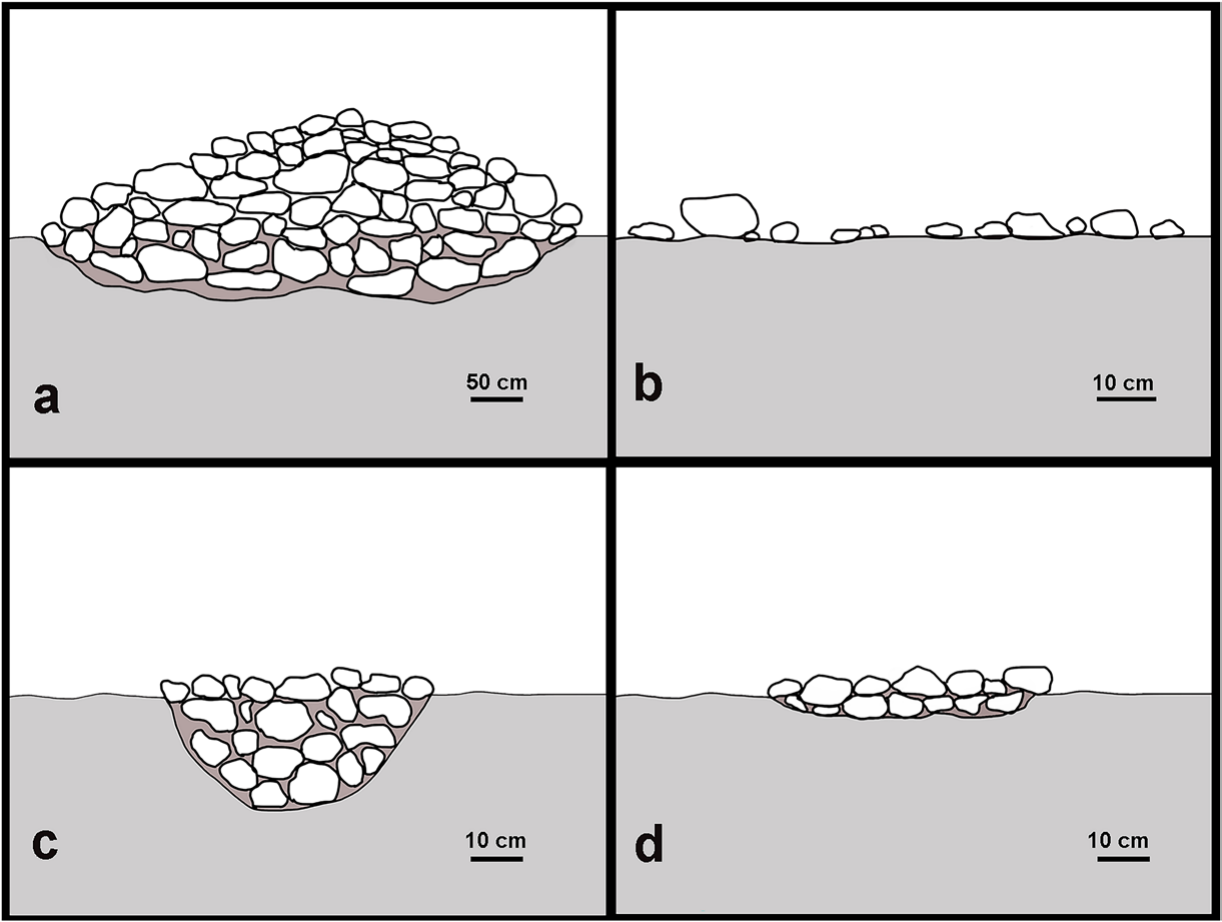
Schematic profile illustration examples of stone structures: ( **a**) burnt mound, ( **b**) scattered stones, ( **c**) pit, ( **d**) heart.

From the 73 items recovered, 59 reported HAS in archaeological contexts, while 35 presented experimental studies mainly focused on the replication of cooking processes. Of the 59 items reporting archaeological contexts, the majority represented archaeological sites in North America (53%) and Europe (36%), followed by few studies in Africa, Oceania, Asia and South America ( Figure 1). Of these, 78% could be assigned to archaeological cultures of the Holocene and the remaining 22% to the Pleistocene. Overall, 39 studies (66%) reported HAS associated to foraging groups, while 10 (17%) reported them among farming societies, and the remaining to a combination of foraging and farming groups due to reports combining multiple sites, chronologies and/or occupations. In addition, only 16 studies (27%) reported HAS in archaeological contexts with ceramic artefacts, while the remaining 43 (73%) either lacked ceramic remains or did not mention their presence. The majority (80%) of HAS documented in the 59 records are associated with some structures, such as burnt mounds, pits, and hearths ( Figure 2).

**Figure 3.**
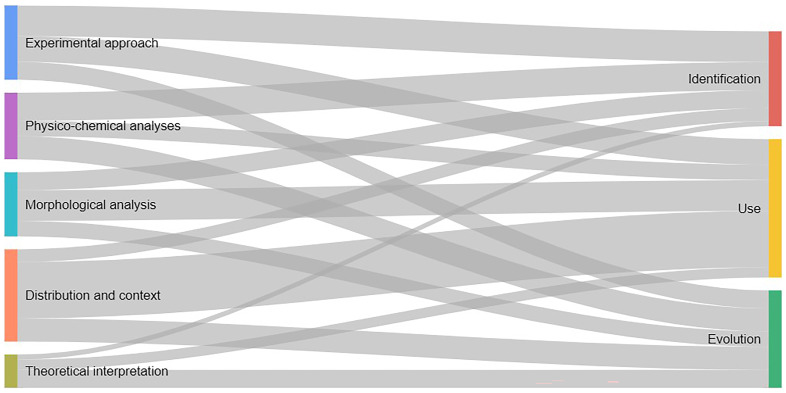
Sankey plot showing the relationship between methodologies applied and archaeological questions about HAS.

Notably, we found up to 32 distinct terminologies used to report HAS, which imposes considerable challenges in comparative analysis and attempts to establish common criteria for the identification of this class of artefacts ( Neubauer, 2018). Moreover, our results indicate a prevalence of a few lithologies as HAS in archaeological records. Specifically, sandstone and quartzite emerge as the predominant group, followed by limestone, granite and quartz. This pattern may suggest deliberate selection of rocks with specific physical and chemical properties, as demonstrated by experimental studies (see below). Furthermore, the results show that the identification and characterization of HAS rely mostly on experimental approaches and physico-chemical analyses, while the functional interpretation of HAS rely primarily on their spatial distribution and contexts ( Figure 3) ( Frison, 1983; Hawkes, 2018; Movius, 1966; Petraglia, 2002; Schaefer-Di Maida, 2022), including the presence of well-defined structures (cooking pits, burnt mound, earth ovens, stone boiling pits, etc).

**Figure 4.**
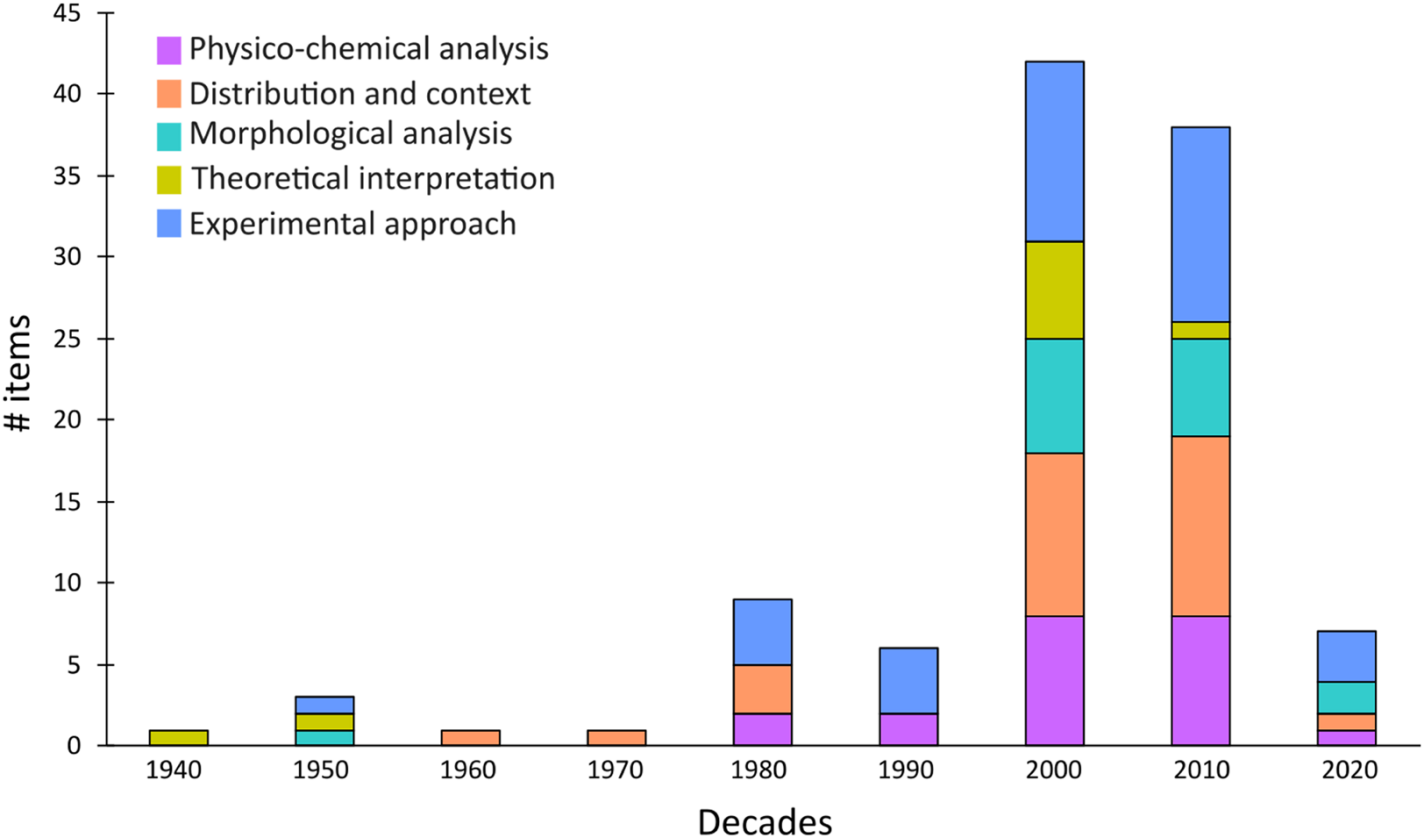
Methodological approach over the 20th and early 21st centuries.

Our analysis revealed a significant shift in the methodological landscape of HAS research over time ( Figure 4). It was not until the 1980s that chemical and physical approaches were applied, along with a marked increase in the frequency of experimental studies. Furthermore, the past two decades have seen an exponential growth in HAS research, combining also different methods. Moreover, identifications through chemical and physical analyses (e.g., digital images, dating technique, magnetic susceptibility), along with morphological observations, and experimental studies have increased in recent years.

## Discussion

### Geographic and chronological distributions of HAS

Our assessment on HAS revealed a strong research bias towards evidence from North America and Europe, as recently reported by Neubauer ( Neubauer, 2024). HAS are documented since the Pleistocene from these regions, attesting that culinary and non-culinary activities using heating stones emerged among foraging groups subsisting on hunting, fishing and gathering ( Petraglia, 2002; Thoms, 2009). We observed considerable gaps in knowledge on HAS for Africa, Oceania, Asia, and South America, which are key geographic areas for assessing the role of heating stones in human evolution, geographic dispersal, early cuisine and diet, and cultural transmission across the globe. We consider these regions as priority areas for future research on prehistoric HAS.

The high frequency of HAS during the middle and late Holocene testifies to the endurance of some practices over long time periods, amid the emergence of new food systems and the introduction of new resources and technologies, such as domesticated plants and animals, and ceramic containers. In North America, HAS have been predominantly reported among “Archaic” groups with foraging subsistence systems ( Griffin, 1967; Sutton, 2015). A few referred to the middle Woodland period ( Custer, 2017; Pagoulatos, 1992; Skibo *et al.*, 2009) and to the Basketmaker II period ( Ellwood *et al.*, 2013), which exhibits evidence of agricultural practices. In Europe, HAS were reported in few Palaeolithic and Mesolithic sites ( Bang-Andersen, 2015; Little, 2014; Movius, 1966; Nakazawa *et al.*, 2009; Pop *et al.*, 2021), although structures containing HAS were extensively documented among farming societies of north Europe from the Neolithic to the Iron Age ( Anthony *et al.*, 2001; Barfield & Hodder, 1987; Eskola *et al.*, 2003; Hawkes, 2018; Lindgaard, 2015; Ó Drisceoil, 1988; O’Kelly, 1954; Petersson, 2013; Schaefer-Di Maida, 2022; Swedberg *et al.*, 2017; Viberg *et al.*, 2013). These structures have been documented as *fulacht fiadh* in archaeological sites in Ireland and as burnt mounds in Britain ( Anthony *et al.*, 2001; Barfield & Hodder, 1987; Hawkes, 2018; Ó Drisceoil, 1988). Their use encompasses culinary practices, as well as burial, domestic and ritual activities (e.g. sweat lodge, feasts) involving large groups of people ( Barfield & Hodder, 1987; Custer, 2017; Hawkes, 2018; Ó Drisceoil, 1988; Petersson, 2013), indicating that HAS may result from cooking and non-cooking activities. Comparable structures were defined as cooking stone pits in the German and Scandinavian literature ( Fretheim, 2009; Schaefer-Di Maida, 2022), suggesting their use predominantly for cooking activities but without certain evidence ( Swedberg *et al.*, 2017).

### Identification and characterization of HAS

One of the most significant challenges in the field of archaeology is the continued uncertainty surrounding the fundamental definitions of these artefacts and the key diagnostic elements for their identification. We also observed a generalised lack of consistency in how HAS are documented and reported, as previously observed by others ( Graesch *et al.*, 2014; Pop *et al.*, 2021), which highlight some of the challenges of studying HAS. For example, Fire-Cracked Rocks (FCR), which describes stones that have been fragmented and shattered by fire in cooking activities, was the most commonly reported term, occuring in 61% of the items ( Black & Thoms, 2015; Custer, 2017; Cutts *et al.*, 2019; Gao *et al.*, 2014; Neubauer, 2018; Stuart & Walker, 2018; Sullivan *et al.*, 2001; Thoms, 2009). This was followed by the terms “heated” and “hot stones” ( Ellwood *et al.*, 2013; Nelson, 2010; Pop *et al.*, 2021; Schaefer-Di Maida, 2022; Thoms, 2008a), along with “burned stones” ( Asfora *et al.*, 2014; Black & Thoms, 2015; Bock *et al.*, 2017; Buonasera, 2005). In addition, the terms “cooking” and “boiling stones” were widely used, implying specific functions in culinary activities ( Buonasera, 2005; Shantry, 2020; Short *et al.*, 2015) and in boiling liquids ( Pop *et al.*, 2021; Shantry, 2020; Short *et al.*, 2015; Thoms, 2008b). Also reported were: “firebroken rocks” (FBR), denoting rocks that exhibit characteristics of thermally induced fractures due to heating and subsequent cooling in water ( Brink & Dawe, 2003); “fire-modified rocks” (FMR), used to indicate rocks that have undergone some anthropogenic modification to improve mechanical features ( Carney *et al.*, 2022; Oestmo, 2013); “thermally altered” and “thermally modified rocks” (TMR), used to indicate stones that present physical transformations, such as fractures, cracking, crazing and discoloration, as a result of exposure to heating and cooling process ( Bentsen & Wurz, 2019; Graesch *et al.*, 2014; Homsey, 2009; Nakazawa *et al.*, 2009; Petraglia, 2002); “culturally-heated rock” (CHR), representing rocks exposed to heat as a result of different cooking methods ( Shantry, 2020), among others. In an attempt to systematise the terminology we recommend the use of HAS when reporting this class of artefact in the absence of information on the artefact-specific functions.

Along with the terminology employed to describe the stones under investigation, we encountered a multitude of wording used in literature to define rock alterations caused by heat and/or fire. This observation has been previously acknowledged by Neubauer ( Neubauer, 2018) who proposed a standardised terminology to help researchers with their description. A list of twelve distinctive patterns related to use alteration of HAS was provided along with three different types of fractures based on size and morphology ( Table 1).

**Table 1.** Heat/fire alteration patterns of HAS according to Neubauer ( Neubauer, 2018).

Use alteration patterns	Fracture types
1. Heat fracture 2. Differential luster 3. Fine crazing 4. Deep surface cracking 5. Exfoliation 6. Pit-lid negative/scar fracture 7. Discoloration 8. Reddening 9. Sooting 10. Oxidised patch 11. Irony oxidation 12. Adhesion	• spall/pseudo flake fragment, small and the size of a flake. • blocky/pseudo core fragment, when the stone is broken along its core. • Pot-lid “flake” fracture, which results from differential expansion causing the detachment of a fragment that appears flat on the dorsal side and convex on the ventral side, with its major thickness in the central part.

Changes in colour represents one of the more intuitive indicators for identifying thermally altered rocks, being a consequence of direct and indirect fire exposure ( Pagoulatos, 2005). These changes are normally associated with a reddening of the exposed rock surface due to thermal alteration of the iron oxides. In some cases, experimental studies involved the evaluation of colour changes after heating processes between experimental and prehistoric stones. The changes are usually evaluated through visual observation, Munsell colour notations and their relative conversion into numerical values, and digital imaging ( Bentsen & Wurz, 2019; Oestmo, 2013; Schaefer *et al.*, 2014). Digital imaging techniques favoured the differentiation between unburned and experimentally burned stones based on their physical appearance ( Oestmo, 2013). Given that visual observation is a subjective criterion that could be affected by the colour perception of the observer, it is crucial not to rely exclusively on colour evaluation, but to complement it with additional techniques, for example petrographic analysis, that could detect microscopic features associated with heat alteration ( Homsey, 2009). Therefore, some researchers advanced the interpretation of archaeological stones throughout experimentation, considering not only discoloration, but other key factors such as fracture rate, fragment type, crazing, pocking, spalling and shapes (e.g. prismatic or platy) ( Custer, 2017; Graesch *et al.*, 2014; Nickels *et al.*, 2001; Pagoulatos, 1992; Pagoulatos, 2005). Moreover, experimental simulations suggested that thermal alterations were less present in stones exposed to short-term re-use, while whitening, cracking and spalling were more common on long-term re-use ( Pagoulatos, 2005). A prolonged exposure to heat might cause cracking in the form of thin crack lines and spalling (the fragmentation of the stones), while sudden heating led to pot lids, crater-like surface depressions ( Cutts *et al.*, 2019; Pagoulatos, 2005). Neubauer ( Neubauer, 2018) also proposed four attributes to determine the reuse of HAS. The absence of cortex along with a high percentage of fracturing pattern could indicate multiple episodes of heating and cooling, while small size and low weight could suggest that the stone has been repeatedly used.

The identification of heated or non-heated stones has also been tested through the use of physical methods. Archaeomagnetic techniques, based on magnetic susceptibility which is greater in burned stones, has proven useful in identifying stones that were heated to at least 200°C ( Barbetti *et al.*, 1980; Bischoff *et al.*, 1984; Gose, 2000; Nickels *et al.*, 2001). This is an important approach for distinguishing *in-situ* oven and griddle stones from those heated in one place and cooled in another ( Nickels *et al.*, 2001; Viberg *et al.*, 2013), as would be the case for stones used in stone boiling or some types of pit steaming and baking ( Abbott & Frederick, 1990; Gose, 2000; Swedberg *et al.*, 2017). Alternative methods employed to test whether the stones were subjected to heating rely on radiometric and luminescence dating methods. Radiometric dating, specifically the ^40^Ar- ^39^Ar technique, involves analysing potassium-bearing minerals from stones to determine if the lithic was heated to a temperature above 500°C for at least 30 minutes ( Gillespie *et al.*, 1989). Among the luminescence-based approaches, optically stimulated luminescence (OSL) was demonstrated to be more suitable compared to thermoluminescence (TL) ( Asfora *et al.*, 2014), including post-infrared infrared (pIRIR) feldspar luminescence ( Pop *et al.*, 2021), due to its reduced material requirement and faster processing time ( Rapp *et al.*, 1999).

### Functional interpretation of HAS

**Figure 5.**
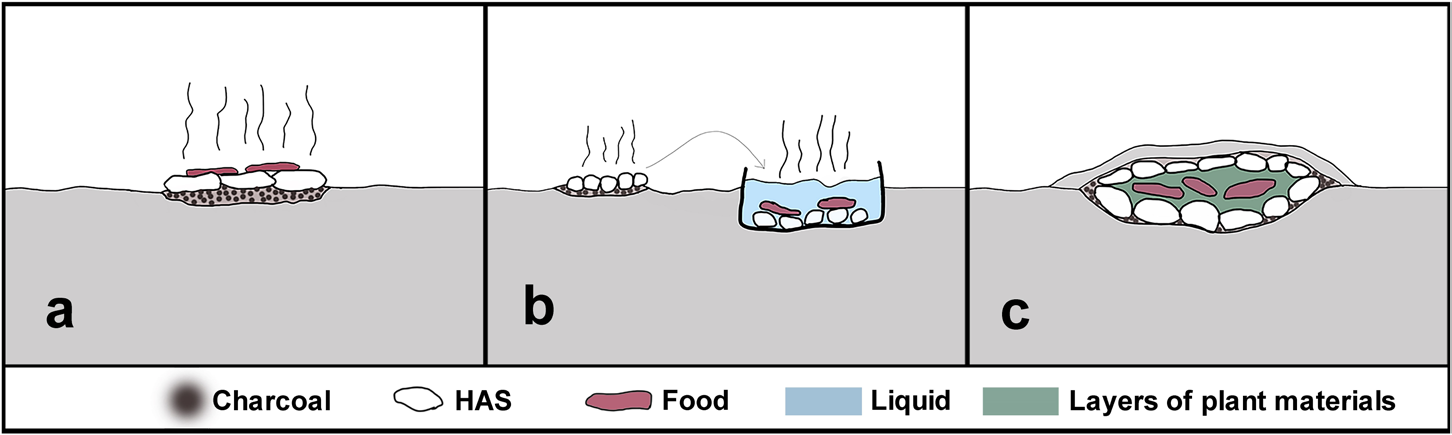
Illustration example of generic cooking techniques: ( **A**) platform hearth, ( **B**) stone boiling technique, ( **C**) earth oven.

Our literature investigation documented a range of cooking techniques that may have involved heating stones, including platform hearth, stone boiling and earth ovens ( Figure 5). These techniques are reported throughout distinct geographic and temporal scales in North America and Europe. Platform hearth, also called rock griddle or roasting pit, is considered an open-air cooking technique consisting of a single layer of rocks upon which a fire is lit. Food can be cooked/dried directly on the rock surface or in a vessel in contact with the flames ( Brink & Dawe, 2003; Custer, 2017; Neubauer, 2018; Thoms, 2007; Thoms, 2008a). Stone boiling technique involves the heating of a liquid that usually is the cooking medium. Stones previously heated in a nearby fire are placed into a container with the cooking liquid, and the stones heat heat the liquid in which the food will be processed ( Brink & Dawe, 2003; Nelson, 2010; Neubauer, 2018; Short *et al.*, 2015; Speth, 2015; Thoms, 2007; Thoms, 2008a). This practice is also called cooking by convection ( Graesch *et al.*, 2014) and can also be used in grease rendering ( Nakazawa *et al.*, 2009; Oetelaar & Beaudoin, 2016) and the preparation of an animal carcass for skinning and removing hair ( Custer, 2017). Earth ovens permit baking food thanks to the moist heat produced in an oxygen-reduced environment, even for a long period of time. They are typically formed by a pit filled with stones heated by a fire and covered with plants and food topped with an additional layer of plant materials and hot rocks, all covered by a layer of earth ( Black & Thoms, 2015; Carney *et al.*, 2022; Custer, 2017; Hawkes, 2018; Homsey, 2009; Leach *et al.*, 2005; Neubauer, 2018; Nickels *et al.*, 2001; Short *et al.*, 2015). A variant of the same structure, termed a steaming pit, is characterised by a small hole in the earthen lid allowing the release of the steam or the addition of water ( Custer, 2017; Driver & Massey, 1957; Hawkes, 2018; Thoms, 2007; Thoms, 2008a).

In addition, an attempt was made to distinguish HAS used in ritual activities from those used for culinary practices ( Lindgaard, 2015). The abundance of HAS in the proximity of a water source, combined with the absence of settlement evidence and food remains, suggested that HAS were likely used for heat and steam production related to potential sweat lodge rituals rather than domestic culinary activities ( Barfield & Hodder, 1987). Moreover, some investigations have assumed that stones used in rituals were subjected to prolonged and intense heat, resulting in a lower density compared to those used for cooking ( Clark & Custer, 2003; Custer, 2017). Although not conclusive, this suggests that density may serve as a proxy for determining the function of HAS.

Cooking practices using heated stones were also likely affected by environmental factors, as some techniques are more or less effective under different climatic and ecological conditions. For example, although stone boiling was widespread among Indigenous groups in North America, its use was limited in areas with scarce fuel material (wood) such as the Arctic and subarctic regions ( Driver & Massey, 1957). Subsequent studies have proposed that stone boiling requires large amounts of fuel and heat conservation capacity to increase the stone temperature for its efficient use ( Nelson, 2010), making it less efficient and more labour intensive in cold regions.

A large body of the assessed literature focused on establishing visual and chemical diagnostic features in order to accurately identify and assess the function of HAS in prehistoric contexts ( Table 2). These approaches typically use both archaeological observations ( Bang-Andersen, 2015; Leach *et al.*, 2005; Little, 2014; Schaefer-Di Maida, 2022; Shiner & Shiner, 1977) and experimental studies ( Backhouse & Johnson, 2007; Brink & Dawe, 2003; Gao *et al.*, 2014; Homsey, 2009; Thoms, 2008a; Wilson & DeLyria, 1999).

**Table 2.** HAS features related to culinary practices.

Cooking technique	Stones features	Key references
Stone Boiling	River cobbles, crenulated fractures, crazing, reddening	( Brink & Dawe, 2003)
	Contraction fractures, crazing, jaggies	( Carney *et al*., 2022)
	Small to medium-sized rocks, lack of carbon staining	( Thoms, 2008a)
	Breakage	( Gao *et al*., 2014)
	Crenulated fractures, angular fragments	( Oetelaar & Beaudoin, 2016)
	Irregular crenulated fractures on the breakage faces and ridges on the inside (wet-cooling contraction)	( Neubauer, 2018)
	Angular profile, low weight	( Petraglia, 2002)
	Core type fragments	( Custer, 2017)
Roasting pit/Griddle	Flat slabs, red oxidation stain on the upper surface, scorching, discoloration, spalling, separation along bedding planes	( Brink & Dawe, 2003)
	Spalling, smooth contours and fairly sharp edges	( Oetelaar & Beaudoin, 2016)
	Boulder to cobble-size stones, spalling	( Carney *et al*., 2022)
Earth oven/ Steaming pit	Flat, convex/concave breakage faces	( Carney *et al*., 2022)
	Carbon staining, medium to large flat rocks, cracking, oxidation	( Thoms, 2008a)
	Flat, convex, or concave breakage faces (dry-cooled expansion fracturing)	( Neubauer, 2018)
	Numerous fractures	( Gao *et al*., 2014)

Expansion and contraction were defined as two main agents of fractures by Neubauer ( Neubauer, 2018) and were related to specific cooking technologies. Expansion breakages occur in stones heated and cooled in hearths. Since the external part is subjected to more heat and expands faster than the inner part, stones that underwent this dry-cooled process (open-air hearths) display flat, convex, or concave breakage faces that are smooth. On the other hand, contraction develops mostly in heated rocks used for stone boiling. During a rapid cooling in water the external part contracts and undergoes a thermal shock, causing irregular fractures on the breakage faces and some ridges on the inside of the stone. While the presence of both features may indicate reuse, another potential sign is the refitting of discarded HAS fragments ( Leesch *et al.*, 2010). Moreover, it is crucial to note that the final location of HAS fragments at the site may not accurately reflect the position of the fire structure where they were last used.

By conducting a series of experimental fire procedures and replicating specific technologies, such as platform hearths, stone boiling, earth ovens and sweat lodges, various studies have established some correlations between the temperature to which the rocks were exposed, the duration of exposure and the number of cracked gravels generated from stones ( Backhouse & Johnson, 2007; Custer, 2017; Ellwood *et al.*, 2013; Graesch *et al.*, 2014; Mercieca, 2000). Hypothetically, an increase in temperature reflects an increase in the number of fractures and with each successive firing, the heated rocks gradually weaken, resulting in a greater quantity of smaller fractured rock. According to that, some authors assumed the use and reuse of rocks based on the dimension of stones, evaluating a pebble to cobble ratio. Larger stones may have been primarily used in food preparation features, while smaller fractured rocks are indicative of prolonged or more intense cooking stone usage ( Carney *et al.*, 2022). Specifically, the stone boiling process required the removal of the stones and their subsequent re-heating, which may lead to a progressive fragmentation due to thermal shock ( Graesch *et al.*, 2014; Shantry, 2020). In his studies Thoms ( Thoms, 2008a) developed several experimental works on the creation and use of stone-cooking facilities. The outcome was that rocks used for simulating earth-oven cooking showed more evidence of cracking, oxidation and increased porosity, than rocks used to simulate the boiling technique.

Our research suggests that mineral composition and physical properties may similarly have played a functional role in heat stone facilities for culinary and non-culinary purposes. Some rocks (and their derived stones) may have been particularly desirable for certain culinary functions, such as quartzite and limestone exhibiting fracture resistance that allows them to break into small and reusable pieces without disintegrating, unlike granite ( Frison, 1983). For example, a common characteristic among many of the recorded lithologies is the prevalence of quartz. This mineral possesses a high refractory property and a unique ability to withstand extremely high temperatures. Its low coefficient of thermal expansion allows it to be repeatedly heated with minimal risk of breakage due to thermal shock. Experimental studies have been instrumental in testing such functional hypotheses. In some studies, the effectiveness of different rock types was investigated in relation to different cooking methods by means of controlled experiments and observations. In contrast with the extensive use of sandstones in different archaeological sites, the experimental study of Bring and Dawe ( Brink & Dawe, 2003) showed that this rock type is particularly fragile, and tends to fracture more readily than fine-grained rocks when reheated. Sandstones also release substantial amounts of sand that may be undesirable during cooking activities ( Brink & Dawe, 2003). Moreover, an experimental study by Ellwood ( Ellwood *et al.*, 2013) demonstrated that limestones are effective for cooking maize using hot-stones (stone-boiling), creating an alkaline cooking environment which improves the availability of important maize proteins. Limestones, however, were considered as not appropriate for boiling liquid substances because of the formation of flakes and powder during repeated heating and immersion in water ( Gao *et al.*, 2014). A similar issue was observed recently for coarse-grained basalts used for the hot stone cooking method. Placing heated coarse-grained basalt stones directly inside a vessel containing water led to the contamination of the liquid with ash and soil, compromising the palatability and edibility of the food ( Langley *et al.*, 2023). On the other hand, coarse-grained rocks (andesite, basalt, diorite, granite, gabbro) appeared to be more resistant to breakage after multiple heating episodes compared to fine-grained stones (schist, quartzite, siltstone, basalt, basaltic andesite). Basalt was the most resistant type, while on the other hand quartzite fragmented into several pieces after a few heatings ( Shantry, 2020; Wilson & DeLyria, 1999).

Organic residues absorbed by or adhered to HAS present valuable information for identifying their culinary function and providing valuable information on subsistence resources. Raman spectroscopy studies, such as those conducted by Short *et al.* ( Short *et al.*, 2015), have observed traces of carbohydrates on archaeological stones, indicating their potential use in plant processing. Additionally, research has demonstrated the feasibility of lipid extraction from archaeological stones, representing a largely untapped source of information into past cuisines ( Buonasera, 2005; Quigg *et al.*, 2001; Skibo *et al.*, 2009). For example, the presence of lipid residues on HAS, likely absorbed into the fractures created by heating, along with the detection of cholesterol in some samples suggested that HAS were used during the processing of animal products ( Skibo *et al.*, 2009). Nevertheless, challenges, including contamination, warrant further research efforts in this direction.

Ethnographic reports of North American Indigenous groups ( Driver & Massey, 1957) attested the use of HAS in earth ovens primarily for processing plant commodities, including roots, bulbs and tubers, along with a frequent use of stone boiling techniques among mobile groups who employed perishable materials such as woven and bark baskets, dugout wood, and animal tissues as containers. Additionally, some structures, such as burned rock middens, are not results of random HAS accumulations but are indicative of recurrently used earth ovens, the size of which is directly correlated with their use frequency and duration ( Mitchell, 2008).

### Social implication

Studies show that HAS carry significant cultural information on regional and local economies, group and population size, and site formation processes ( Hawkes, 2018; Langley *et al.*, 2023; Petraglia, 2002). The presence, frequency and size of structures containing HAS can offer clues about social dynamics. These include technological advances ( Speth, 2015), such as the evolution of stone boiling to animal bone grease rendering ( Nakazawa *et al.*, 2009; Oetelaar & Beaudoin, 2016), as well as social organisation, since the increased effort required to build and maintain some structures, along with intensification of food procurement and processing ( Thoms, 2008b; Thoms, 2009), suggests possible cooperation between different groups of people ( Fretheim, 2009; Hawkes, 2018). Additionally, these archaeological features offer evidence of domestic and ceremonial/ritual activities, including festive occasions involving large groups of people participating in cooking activities ( Hawkes, 2018; Mitchell, 2008; Petersson, 2013; Swedberg *et al.*, 2017) along with different uses beyond culinary applications ( Hawkes, 2018; Ó Drisceoil, 1988), hinting at the versatility of HAS in ancient societies. In some cases, the appearance of HAS within specific structures (e.g. earth ovens, burnt mounds) corresponds with settlement changes, particularly in terms of assessing residential mobility ( Mitchell, 2008) and site occupation patterns ( O’Kelly, 1954; Petraglia, 2002). Social activities such as feasting may have influenced interactions between people, raising the sense of community, along with modifications in long-term production organisation into a more organised management of goods ( Fretheim, 2009; Mitchell, 2008).

Experimental cooking simulations, including the reconstruction of hearths and the replication of stone boiling, have demonstrated the effort and resources required to use and manage fire structures. In some cases, the amount and size of HAS can provide insight into the intensity and duration of human activity at a site, along with the duration of the site occupation ( Jensen *et al.*, 1999; O’Kelly, 1954). By comparing experimental results and archaeological features, researchers suggested that some structures were likely used for a short period of time ( Nickels *et al.*, 2001; Petraglia, 2002). Furthermore, the rapid breakage of HAS with repeated use implies the reduction of their efficiency as boiling stones, thus their discard ( Jensen *et al.*, 1999). For example, Sorensen and Scherjon ( Sorensen & Scherjon, 2018) developed a computer-based model simulating how various factors influence the abundance of HAS found in archaeological sites, demonstrating that the abundance of HAS can be affected by the size and frequency of fires. On the other hand, the lack of burned HAS in an accumulation of stones could suggest minimal human use of these objects ( Bock *et al.*, 2017). 

The reuse of HAS within the settlement represents an important indicator of human resource management. These lithics can undergo multiple cycles within fire structures before becoming functionally ineffective and subsequently discarded. Burned rock middens serve as an example of structures composed of several discarded HAS ( Black & Thoms, 2015; Kelley & Campbell, 1942). Archaeomagnetic techniques hold promise for distinguishing whether HAS were heated *in-situ* or elsewhere. This could lead to better understanding the arrangements and the construction processes of fireplaces in a settlement, along with revealing the duration of heating and the maximum temperature reached ( Gose, 2000; Nickels *et al.*, 2001). These analyses may reveal that HAS were previously part of the cover of a fireplace, being subsequently removed and placed upside down after final cooling.

Finally, dating techniques are powerful tools for placing artefacts and settlements in a specific timeframe. In the context of our research, HAS have been analysed through different dating methods such as TL, OSL and phototransferred thermoluminescence (PTTL) to assess a timespan in which the stones were heated and presumably used in fire-related activities ( Anthony *et al.*, 2001; Eskola *et al.*, 2003; Okkonen, 2010; Rhodes *et al.*, 2009).

## Concluding remarks

This review reveals significant research gaps concerning the study of HAS found in prehistoric archaeological sites. Despite decades of research, it is surprising that they continue to be generally overlooked and systematic studies on them are scarce given their potential to provide insights into various aspects of past human behaviour, including culinary practices, domestic activities, and rituals. Thus a more in-depth consideration of these artefacts is warranted. HAS are associated with a range of heating facilities, for culinary and non-culinary purposes. They have an extensive geographical and chronological distribution in North America and Europe, but notable gaps exist in our knowledge for other regions preventing us from understanding their role in human prehistory, including global dispersal and adaptation to distinct climates and ecological contexts. Identification and characterization of HAS remain a major challenge in archaeology, and should be addressed through multidisciplinary approaches that describe and quantify a range of features, including stone composition (mineral identification), physical properties (size, shape, structure, texture, mechanical and thermal characteristics) and alterations (fragmentation, fractures, discoloration, sooting, reddening), chemical characterization of adhered and absorbed residues along with the spatial distribution, and *in-situ* contextual information (presence of archaeological features). Moreover, the field faces additional persistent challenges, including a lack of standardised terminology for describing HAS and their relative heat alteration, along with the need for a more systematic approach for documenting, and the difficulty in distinguishing between culinary and non-culinary uses.

We acknowledge that our literature review has some inherent limitations, particularly concerning the volume of work not considered here, represented by non-English and grey literature. It is our opinion that integrating these other literatures is more feasible in regional literature reviews or when dealing with specific chrono-cultural phases. Nevertheless, the study emphasises the potential of HAS research to provide insights into prehistoric culinary practices, cultural transmission, and social dynamics. It calls for more comprehensive and interdisciplinary approaches for advancing our understanding of heat-altered stones, their function and significance in prehistoric practices, leveraging their heritage values and highlighting their untapped potential to the wider archaeological community.

## Data Availability

Zenodo. Outputs of the systematic literature review on Heat-Altered Stones. DOI: https://doi.org/10.5281/zenodo.14005823 ( Cantelli, 2024a). This project contains the following underlying data: Outputs of the systematic literature review on Heat-Altered Stones (The document provides a structured overview of key variables extracted from each record). Data are available under the terms of the Creative Commons Attribution 4.0 International license (CC-BY 4.0). ***Reporting guidelines*** Zenodo. PRISMA checklist and flow diagram. Diamonds in the rough - reconsidering the scientific and heritage value of heat-altered stones in Prehistoric Archaeology, DOI: https://doi.org/10.5281/zenodo.14054789 ( Cantelli, 2024b). This project contains the following extended data: PRISMA checklist and flow diagram. (The files detail the PRISMA checklist and flow diagram relative to the present systematic literature review). Data are available under the term of the Creative Commons Zero v1.0 Universal (CC0).
