# Supplementary material for: Diamonds in the rough - reconsidering the scientific and heritage value of heat-altered stones in prehistoric archaeology through a systematic literature review
Source: Open Res Eur. 2024 Nov 27;4:254. [Version 1] doi: 10.12688/openreseurope.18837.1 (PMC11803202; doi:10.12688/openreseurope.18837.1)
Supplement: Peer review history

# Reviewer report: Reviewer response for version 1

**DOI:** 10.21956/openreseurope.20385.r47719

**Published:** 2024-12-17

**Authors:** Eduardo Paixão

**Affiliations:** 1 TraCEr, laboratory for Traceology and Controlled Experiments, MONREPOS Archaeological Research Centre and Museum for Human Behavioural Evolution, Neuwied, Germany; 2 ICArEHB - Interdisciplinary Center for Archaeology and Evolution of Human Behavior. University of Algarve (Ringgold ID: 450639), Faro, Faro District, Portugal

First of all, I would like to congratulate the authors for their research. This paper focuses on a literature review of research on heat-altered stones, a topic that, as pointed out by the authors, is often neglected.

 In my opinion, this paper makes a significant and solid contribution to this field by offering a global overview of the existing publications on the subject.

 However, this study contains some limitations that are recognized by the authors. First, the exclusion of grey literature, which might contain valuable information, is a limitation. Secondly, the challenges related to the indexing of certain publications mean that some relevant works may naturally have been omitted from the search results. Nevertheless, in my opinion, these limitations are well recognized and justified by the authors throughout the text.

 Another aspect that in my opinion could be further developed is the information and discussion about the ethnographic studies since it is superficially approached and focused on North America.

 A particularly strong point of the paper is its emphasis on the challenges with the lack of systematisation in terminology, a problem that is often recognised across multiple fields of use-wear analysis.

 This study also shows the regional gaps in the knowledge about these technologies, pointing out the importance of more functional studies.

 In sum, this study is important to bring well-deserved attention to a category of artefacts that often preserve crucial information about past behaviours, and by presenting a comprehensive state-of-the-art and can support further research in this field.

If any, are all the source data and materials underlying the results available?

Yes

Is the work original in terms of material and argument?

Not applicable

Is the work clearly and cogently presented?

Yes

Is the argument persuasive and supported by evidence?

Yes

Does the research article contribute to the cultural, historical, social understanding of the field?

Yes

Does it sufficiently engage with relevant methodologies and secondary literature on the topic?

Yes

Reviewer Expertise:

Paleolithic Archeology; Use-wear analyses; Lithic technology

I confirm that I have read this submission and believe that I have an appropriate level of expertise to confirm that it is of an acceptable scientific standard.

# Reviewer report: Reviewer response for version 1

**DOI:** 10.21956/openreseurope.20385.r47712

**Published:** 2024-12-10

**Authors:** Marcelo Cardillo

**Affiliations:** 1 Multidisciplinary Institute of History and Human Sciences, Faculty of Philosophy and Letters, University of Buenos Aires, Buenos Aires, Argentina

The article *Diamonds in the Rough - Reconsidering the Scientific and Heritage Value of Heat-Altered Stones in Prehistoric Archaeology Through a Systematic Literature Review* provides a comprehensive review of heat-altered stones in prehistoric archaeology. This work highlights the characterization of the main research lines associated with this type of material evidence, as well as the challenges linked to its study, such as determining its functional aspects, its role within prehistoric societies (e.g., in analyzing food processing techniques, spatial use, or symbolic significance). The authors rightly conclude that the study of heat-altered stones remains an underexplored field, facing significant challenges such as establishing shared methodological guidelines for their analysis.

 In our work related to the topic addressed in this article, we have followed a taphonomic approach to study the effects of natural wildfires on rocks. I take the liberty of suggesting our recent article, which may be of interest to the authors:

 [Carranza, E., & Cardillo, M. (2024).Ref-1].

 One aspect of interest that may not be present in the mentioned research is the role of natural and post-depositional processes in the formation of heat-altered rock accumulations, as well as the changes that alteration variables undergo over time due to mechanisms such as water , abrasion, and trampling, among others. These changes can obliterate or complicate their study, recognition, and interpretation.

If any, are all the source data and materials underlying the results available?

Yes

Is the work original in terms of material and argument?

Yes

Is the work clearly and cogently presented?

Yes

Is the argument persuasive and supported by evidence?

Yes

Does the research article contribute to the cultural, historical, social understanding of the field?

Yes

Does it sufficiently engage with relevant methodologies and secondary literature on the topic?

Yes

Reviewer Expertise:

Lithic technology, experimental methos and applied statistics

I confirm that I have read this submission and believe that I have an appropriate level of expertise to confirm that it is of an acceptable scientific standard.

## References

[1] : Burned Lithics: An Experimental Approach to Characterize the Effects of Fire on the Lithic Archaeological Record of Northern Patagonia, Argentina. *Lithic Technology* .2024; 10.1080/01977261.2024.2425876 1-18 10.1080/01977261.2024.2425876
